# Effect of owner-controlled acaricidal treatment on tick infestation and immune response to tick-borne pathogens in naturally infested dogs from Eastern Austria

**DOI:** 10.1186/1756-3305-6-62

**Published:** 2013-03-09

**Authors:** Michael Leschnik, Andrea Feiler, Georg G Duscher, Anja Joachim

**Affiliations:** 1 Small Animal Clinic, Department for Companion Animals, University of Veterinary Medicine Vienna, Veterinaerplatz 1, Vienna, A-1210, Austria; 2 Institute of Parasitology, Department for Pathobiology, University of Veterinary Medicine Vienna, Veterinaerplatz 1, Vienna, A-1210, Austria

**Keywords:** Tick infestation, Tick-borne pathogens, Immune response, Acaricides, Efficacy

## Abstract

**Background:**

Tick-borne infections resulting from regular tick infestation in dogs are a common veterinary health problem all over the world. The application of repellent and acaricidal agents to prevent transmission of pathogens is a major protection strategy and has been proven to be highly effective in several trials under laboratory and natural conditions in dogs. Despite such promising results, many dog owners still report tick infestation in their dogs although acaricidal agents are used. Information about the current infection status and changes of the infection status regarding tick-borne diseases (TBD) in dogs treated by the owner’s controlled acaricide application is lacking.

**Methods:**

In this study 30 dogs were each treated with permethrin, fipronil + S-methoprene, or served as untreated controls. Application of the acaricide was performed by the owner who decided when and how often to use the spot on preparation. Over a period of 11 months, dogs were clinically examined and sampled for antibody responses against *Babesia canis*, *Anaplasma phagocytophilum*, *Borrelia burgdorferi* s. l., and TBE virus before the study started, 6 months later and at the end of the investigation period.

**Results:**

The permethrin acaricide was applied on average 3.40 times within the examination period, whereas the fipronil + S-methoprene medication was applied 3.03 times. Approximately 2/3 of all dogs, independent of the group, had a positive immune response to one or more pathogens. Three dogs developed clinical symptoms of canine babesiosis, all other dogs remained healthy. Individual number of ticks per dog or number of infections per dog did not correlate with the application rate, and the number of ticks per dog did not influence the number of infections per dog. As owners did not apply the acaricides regularly no influence on the number of infections could be documented although the number of ticks was clearly reduced by the application of the spot-on drugs.

**Conclusions:**

Clinical disease in dogs exposed to tick-borne pathogens is rare, although a humoral immune response reflecting infection is common. More educational training for dog owners is necessary to make the application of acaricides effective regarding the prevention of tick-borne diseases.

## Background

For the canine population in Central Europe, risk assessment studies on tick-borne diseases cover the investigation of canine tick infestation [1], the prevalence of pathogens in infested ticks [2], canine immune responses to tick-borne pathogens [3] and symptomatic disease in dogs [4]. Four major tick-borne diseases have been described in Central Europe in dogs in the last decades: canine babesiosis caused by *Babesia canis canis*[5], canine granulocytic anaplasmosis (*Anaplasma phagocytophilum*; [6]), canine borreliosis (*Borrelia burgdorferi* sensu lato; [7]), and tick-borne encephalitis (TBE; Flavivirus; [8]). Strategies to prevent vector-borne diseases in dogs include vaccination against some agents and the application of repellent and acaricidal agents. Several synthetic drugs have been evaluated for their efficacy to prevent tick infestation (repellent effect) and their acaricidal properties [9]. The efficacy of acaricides for preventing infection depends on the repellent competence, time until tick killing, and the minimal transmission time of the pathogens.

Several acaricidal agents have been tested for their repellent and acaricidal properties, mainly under laboratory conditions, but also in field trials [10], evaluating also the efficacy against immature stages of the ticks. Permethrin 65% has been shown to have a higher efficacy in terms of repellency and tick killing efficacy than fipronil 9.7% against adult *Ixodes ricinus* under laboratory conditions in dogs [11]. In the field trial by Otranto *et al.*[10], a combination of imidacloprid 10%/permethrin 50% or fipronil 10%/(2)-methoprene 12% was used, resulting in a significantly different efficacy against immature stages of *Rhipicephalus sanguineus* at day 28 post-treatment (98.52% versus 72.40%). Both preparations could protect dogs against tick-borne infection by *B. burgdorferi* or *A. phagocytophilum* in an experimental setting as no dog seroconverted after imidacloprid/permethrin and only 1 out of 8 dogs seroconverted after fipronil [12]. Imidacloprid 10%/permethrin 50% was also very efficient (>95%) in protecting a dog population from exposure to *Ehrlichia canis*[13]. A combination of fipronil, amitraz, and (S)-methoprene was shown to efficiently (86%) block transmission of *B. canis canis* by *D. reticulatus* and to protect all dogs from clinical signs [14]. Apart from spot on formulations, an imidacloprid/flumethrin collar has also shown high efficacy against tick infestation [15] and prevention of transmission of *Babesia canis* to dogs [16].

Transmission time of pathogens from the tick to the host has been evaluated mainly for adult ticks under laboratory conditions. Transmission of *A. phagocytophilum* occurs within 24 hours of tick feeding [17,18]. For *B. burgdorferi* s. l. transmission time has been proven starting at the earliest after 16.7 hours of tick attachment to gerbils [19]. Most authors state that 24 to 48 hours of transmission time increases the probability of infection significantly [9]. For the transmission of *B. canis canis* from *D. reticulatus* ticks to dogs it requires more than 48 hours of feeding, except for male *Dermacentor* ticks which repeatedly feed on different individual hosts [18]. TBEs virus has been shown to be transmitted immediately after tick feeding starts as the virus is located in the salivary glands of the infected tick [20].

Despite the proven efficacy of acaricidal agents, many dog owners still report tick infestation in their dogs and high antibody prevalence regarding tick-borne pathogens can be found in dog populations [3,8,21,22], indicating that many dogs are still insufficiently protected from pathogen transmission.

The aim of this study was to evaluate the effect of spot on acaricides, applied by the dog owner, on tick infestation and the immune response to tick-transmissible pathogens in naturally infested dogs under field conditions.

## Methods

### Animals

Ninety clinically healthy dogs, 58 females, 32 males, of different breeds at the age of 6 months to 13 years were included in this study, conducted in 2010. All dogs were living in a rural area known to be endemic for the main vectors (*Ixodes ricinus, Dermacentor reticulatus*) and the four tested pathogens. The area is located in the eastern part of Austria, defined by the four coordinates (N48°6’54”E16°42’9”; N48°17’56”E16°49’49”; N47°55’2”E16°36’50”; N47°51’51”E16°50’56”), and covers about 192 km^2^. Animals were allocated to three groups of 30 animals each. The group “Permethrin” was treated with a commercially available acaricide/repellent (permethrin, Exspot®, Intervet GmbH, Austria); the group “Fipronil” was treated with an acaricide (fipronil + S-methoprene, Frontline® spot on, Merial, France); the group “Untreated” remained without treatment. In the untreated group 2 dogs were euthanized unrelated to tick-borne diseases before the end of the study, in the other groups one dog was excluded each due to poor owner compliance. The acaricidal products were available for free for the study participants. The owners applied the treatment at his/her decision to reflect the actual way the dog would be protected under natural conditions. Owners were also asked to report the treatment and signs of disease such as, fever, anorexia, lameness, or neurological symptoms. The experiments were approved by the institutional ethics committee, (University of Veterinary Medicine Vienna) and the Austrian Ministry for Science and Research (GZ68.205/25-II/10b/2010).

### Sampling

Dogs were taken for a walk daily in an area known to be endemic for ticks and examined for tick infestation by their owners immediately afterwards for an examination period of eleven months (February – December). Only attached and feeding ticks were sampled. Blood samples (serum and EDTA blood samples) were collected three times from each dog, before the examination period (January), 6 months later and at the end of the trial.

### Clinical examination and hematology

At each sampling dogs were clinically examined for signs of acute disease. Hematocrit, total protein, creatinine, alanine aminotransferase, total leukocyte counts and thrombocyte counts were determined to detect subclinical diseases or infections.

### Serology

Blood samples were tested for antibodies against *A. phagocytophilum* (IFAT, MegaScreen FLUOANAPLASMA®, MegaCor Diagnostic GmbH, Austria; cut off titer 1:50), *B. canis* (IFAT, MegaScreen FLUOBABESIA®, MegaCor Diagnostic GmbH, Austria; cut off titer 1:20), and the Tick-Borne Encephalitis (TBE) virus (ELISA, Enzygnost®; Dade Behring, Germany; cut off: 20 IU). Antibodies to *B*. *burgdorferi* s. l. were tested by Western Blot (MegaBlot® IgG, MegaCor, Diagnostic GmbH, Austria) according to the method described by Leschnik *et al.*[3].

A positive immune response to *A. phagocytophilum* or *B. canis* was defined as seroconversion or a two-fold titer increase in the Indirect Immunofluorescence Assay. The cut-off for the TBE-ELISA was 20 IU and a positive immune response was defined as seroconversion or a rise of 20 IU. The *Borrelia* Western Blot bands for p100, BmpA, OspC, and 18 kDa were defined as highly significant (5 points each), bands for 58 kDa and OspA counted as 3 points, and the band p41 with low significance 1 point. Blots with a total score of 0–5 points were defined as negative, 6–9 points resulted in equivocal, and > 10 points positive. A positive immune response was defined as an increase of the total score from negative to positive or an increase of band intensity in two highly specific bands.

### PCR

PCR for *B. canis* and *A. phagocytophilum* was carried out for all dogs at the first and the last sampling time to test for chronic infections without canine immune response (first sampling) and acute infections not detectable by serology (third sampling).

The *B. canis* PCR was carried out in a 25 μl reaction mixture containing PCR buffer, 0.1 mM of each dNTP, 0.01 μM of each primer, 7.5 mM magnesium chloride and 0.5 U of GoTaq® polymerase. The thermal cycling profile was 94°C for five minutes followed by 37 cycles at 94°C for 30 seconds, 56°C for 30 seconds and 72°C for 60 seconds, with a final extension step of 72°C for seven minutes. The PCR primers were based on Zahler *et al.*[23].

The PCR for detection of *A. phagocytophilum* was carried out in a 20 μl reaction mixture containing PCR buffer, 0.1 mM of each dNTP, 0.0125 μM of each primer, 7.5 mM magnesium chloride and 0.5 U of GoTaq® polymerase. The thermal cycling profile was 94°C for five minutes followed by 30 cycles at 96°C for 15 seconds, 66.8°C for 45 seconds and 72°C for 60 seconds, with a final extension step of 72°C for seven minutes. The 16 S rRNA PCR which yields in a 619 bp fragment was done with the primers: Ehr_u_for: 5` - GTT TGA TCC TGG CTC AGG A(C; T)(A,G,T) AAC G - 3`, and Ehr_ERB2_rev: 5`- CTC TCC CGG ACT CTA GTC TGG C - 3`.

### Statistics

Statistical analysis was carried out using SPSS v. 17 (SPSS Inc., Chicago, Illinois). All data sets were tested positive for Gaussian distribution by Kolmogorov-Smirnov test. A logistic regression analysis was performed to demonstrate the increasing probability of a tick-borne infection regarding the ongoing exposure due to aging in the dog population. Pearson’s rank correlation coefficient (ρ) was calculated for the parameters ‘total number of ticks per dog’ and ‘total number of infections per dog’ in relation to the number of acaricide applications. An infection was considered in case of a positive humoral immune response during the study period. Correlations were considered significant when p ≤ 0.05 (two-sided). To compare the number of infections in the three groups a Kruskal-Wallis test was performed.

## Results

### Clinical examination and hematology

All dogs alive were in good health at the three examination days and blood parameters did not reveal any relevant disease that would have excluded the dogs from the study or indicate tick-borne disease.

### Number of treatments applied

In the permethrin group the acaricide was applied on average 3.40 times (range 0–9) within the examination period, in the fipronil group the medication was applied 3.03 times (range 2–6). In winter the permethrin was applied altogether 7 times and fipronil once , in spring 39 and 44 times, in summer 28 and 27 times, and in autumn 27 and 20 times, respectively. A low frequency application (1–3 times) was performed by 44 dog owners; a more seasonal application (4–6 times) by 14 owners, and only 2 dog owners used the acaricide regularly (7 and 9 times).

### Tick infestation

Within 11 months a total of 684 attached ticks were collected. From the dogs in the untreated group a total of 331 ticks were sampled, from those in the permethrin group 155, and from those in the fipronil group 214 ticks.

### Serology and PCR

#### A. phagocytophilum

At the first sampling, 38/90 dogs (42.2%) had antibodies against *A. phagocytophilum*. By the end of the study (third sampling) 48/86 dogs (55.8%) were seropositive. Of the 86 dogs that were available for all three samplings, 25 (29.1%) showed a seroconversion or titer increase. PCR for *A. phagocytophilum* was negative in all samples tested.

#### B. canis

Nine out of 90 dogs (10%) were seropositive for *B. canis* at the beginning of the study; five of them had a history of clinical babesiosis and a positive titer at the first sampling. Four dogs were seropositive at the first sampling without any history of clinical babesiosis. At the end of the season 14 out of 86 samples (16.3%) were positive with in total 5 dogs showing seroconversion and one dog had a rising titer over the entire period. Three dogs were diagnosed with acute babesiosis, including detection of piroplasms in blood smears by a private veterinarian and all showed a specific immune response.

Six of the 86 animals (6.9%) available for the complete study had acquired an infection during the year. None of the blood samples tested positive for *B. canis* by PCR.

#### B. burgdorferi

At the beginning of the study a positive immune response to *B. burgdorferi* s. l. was detected in 39 out of 90 dogs (43.3%). The seroprevalence increased to 45.6% in spring and decreased to 37.2% at the end of the study. In total 14 dogs had been vaccinated (Merilym®, Merial, Lyon, France) before the study started according to their owners, 11 of which were positive in the first sampling. The other 28 seropositive dogs had never received an anti-*Borrelia* vaccination according to their owners. Of the dogs that were not vaccinated during the study, 20 animals (23.2%) seroconverted or had an increasing immune response during the study, indicative of infection.

### TBE virus

A positive immune response to TBE virus was detected in 8 dogs at the beginning of the study; one of these became negative during the summer and 3 dogs had a stable detectable immune response throughout the year, one dog was lost for follow up, and 3 dogs were infected again (rising titer). In all, 10 dogs (11.6%) showed an immune reaction to TBE virus, indicative of contact, and the majority of infections (n = 8) was detected at the last sampling. Only one dog tested positive in the second sample. At the end of the season 12 samples were positive.

### Infections during the year

During the sampling period 61 immune reactions to any of the four tested pathogens were recorded, 30 in spring and 31 in autumn (Table 1). These infections were detected in 47 dogs with 35 single infections, 10 dogs with double infections and two dogs that became infected with three different pathogens (Table 2). The calculated risk of becoming infected with *A. phagocytophilum* was 29.1%, with *B. canis* at 6.9%, *B. burgdorferi* s. l. 23.2%, and with TBE-virus 11.6%. The overall risk of becoming infected with one or more of the investigated pathogens within the sampling period was 54.0%.

**Table 1:** Number of dogs with seroconversion or rising titer during the investigation period / pathogen

**Pathogen**	**Total**	**Spring**	**Autumn**	**Permethrin**	**Fipronil**	**Untreated**
*A. phagocytophilum*	25	12	13	11	6	8
*B. canis*	6	3	3	2	3	1
*B. burgdorferi* s. l.	20	13	7	7	8	5
TBEV	10	2	8	2	4	4
total	61	30	31	22	21	18

**Table 2:** Number and pathogens of double and triple infections during the investigation period

**Pathogens**	**Number**
*A. phagocytophilum* + TBEV	1
*A. phagocytophilum* + *B. burgdorferi* s. l.	2
*B. canis* + *B. burgdorferi* s. l.	3
*B. canis* + TBEV	1
*B. burgdorferi* s. l. + TBEV	3
*A. phagocytophilum* + *B. canis* + *B. burgdorferi* s. l.	1
*B. canis* + *B. burgdorferi* s. l. + TBEV	1

### Tick infestation, acaricide application, and infections

There was no correlation between tick infestation and infection in 90 dogs (ρ = 0.021; p = 0.843). Even in those cases when owners sampled ≥ 20 ticks per year from their dogs, only 5/9 had an infection response. The correlation between the total number of ticks per dog and the number of acaricide applications was not significant in either treatment group (ρ = 0.174, p = 0.358 for the permethrin group; ρ = 0.138, p = 0.467 for the fipronil group). Similarly, the correlation between infection and the number of acaricide applications was not significant for the permethrin group (ρ = −0.107, p = 0.575) or in the fipronil group (ρ = 0.062, p = 0.745).

**Figure 1:**
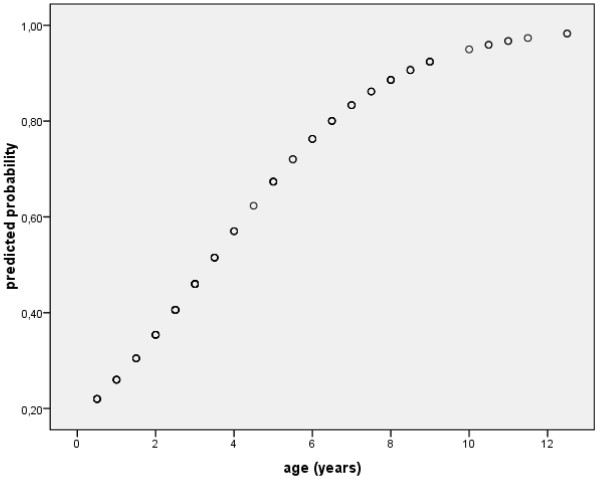
**Correlation of positive humoral immune response and the age of dogs.** The graph displays the predicted probability for one or more positive antibody test results in the 90 dogs regarding the dog’s age at the beginning of the study. When dogs within this certain population reach the age of 3.4 years, there is a 50% chance for those dogs to have a positive antibody test result. At the age of 8.3 years the probability rises up to 90%.

No significant difference could be detected between the numbers of infections in the three groups (p = 0.568, see also Table 1). The predicted probability for a tick-borne infection regarding the age of a dog approximates to 100% within 12 years (50% probability at 3.4 years and 90% probability at 8.3 years; Figure 1).

## Discussion

Dogs are a common target of tick-borne diseases because of their specific natural behavior of moving through the vegetation during walking, leading to a high incidence of tick infestation. Intense tick infestation in dogs might be assumed to result in a high number of infections, although this could not be confirmed in this study. Only five of the nine most highly infested dogs showed a humoral immune response during the year, which might be explained by a weak individual immune response. Maybe also an above-average alertness and ability of those nine dog owners to detect and remove ticks reduced the infection rate when ticks were removed before transmission occurred. On the other hand, dogs without confirmed tick infestation also seroconverted during the study period. Consequently, the total number of tick per dog and the detection of infections with tick-borne pathogens in the examined dogs did not correlate. Although unexpected, the number of missed ticks and the undetermined number of attached but dead ticks, unable to transmit especially *B. canis* and *B. burgdorferi*, for which transmission starts after a feeding time of more than 24 hours, may be the reason for this result.

Irrespective of the total number of ticks per dog, a high risk for contracting a tick-borne infection was demonstrated; according to the presented data, dogs have a high risk to become infected with at least one pathogen during their life. Consequently it is mandatory to apply suitable and effective prophylactic measures against tick infestations and tick-borne infections in dogs, starting early in life.

The risk for the examined dogs to become infected by one of the tested pathogens ranged from 6.9% (*B. canis*) to 29.1% (*A. phagocytophilum*), the total risk was calculated to be 54.0%. This high number is in contrast to the low number of clinical symptoms (three cases of canine babesiosis) in these dogs, even in multiply infected dogs (Table 2), leading to the conclusion that symptomatic tick-borne diseases have a low incidence.

The seroprevalences in the dogs in this study are in the range of other studies from Central Europe for TBE virus (25% in Austria; [8]), *B. canis* (5.7% in Hungary; [21]), *B. burgdorferi* (28.6% in the Czech Republic; [24]), and *A. phagocytophilum* (56.5% in Austria; [22]), so that the selected area of investigation can be considered as representative for a rural Central European location.

PCR examinations were all negative for *B. canis* and for *A. phagocytophilum*. This is not surprising as the first and the third examination were performed in winter, when tick infestation is rare and therefore also the presence of pathogens in the dog’s blood during acute infection was not expected. PCR of blood samples from 3 dogs with acute babesiosis could not be performed as private veterinarians only sent stained blood smears for verification.

There was no correlation between the number of acaricide applications and infection, although a reduction of transmission events is the major reason to apply such preventive measure [13,14]. The highest frequency of immune responses in the dogs were found for *A. phagocytophilum* which is transmitted within the first 24 hours of infestation and *B. burgdorferi* s. l. which has the highest prevalence in ticks [25]. This outlines the major requirements for spot on preparations: the substances should repel ticks to avoid infestation in large numbers and it should kill the ticks as early as possible, best even before the tick starts feeding.

Spot on application and the type of drug did not influence the total number of infections per dog, as there was no significant difference of this parameter in the three groups. Between permethrin and fipronil + S-methoprene no influence on the total number of infections per dog could be found, although Endris *et al.*[11] documented a significantly different efficacy in terms of reducing tick infestation, which has also been confirmed in this study.

When testing the efficacy of acaricidal drugs under natural conditions one has to consider several possible influences: the owner’s ability and alertness to apply the spot on to the skin, to search and remove ticks, and different environmental and behavioral conditions for the dogs, such as walking distance and areas with different tick densities, as well as exposure to sunlight or water, including swimming and rain [26]. Both permethrin and fipronil + S-methoprene are described as efficacious in reducing infestation with *Rhipicephalus sanguineus*, the brown dog tick, under natural conditions, although the effect was less than 100% especially against nymphs [13]. Poor owner compliance in regular application and inappropriate application of the spot on may be reasons for the poor performance of the two drugs in this study, as the application rate in this study was low (on average 3.03 to 3.40 treatments per year), and many owners reported that they applied the spot on when ticks were already observed. Poor owner compliance has also been mentioned and summed up by Stanneck *et al.*[27]. In the current study 2/3 of dog owners were told to use a spot on acaricide, and they applied it in different frequencies, which is comparable to a study where 74% of owners used a tick and flea control product and only 61% of those used these products year around [28].

To overcome this irregular application, caused by non-compliance of the owners, leading to this poor protection status, long lasting measures like slow release collars could be a solution. Acaricidal collars provide high efficacy in terms of preventing tick infestation [15] and pathogen transmission [16] and the effect lasts several months compared to several weeks of spot on formulation with similar efficacies [29]. The repellent efficacy of an imidacloprid/flumethrin collar was demonstrated to be faster than the minimal transmission times for *Borrelia* spp. and *A. phagocytophilum*[15], which is comparable to certain spot on formulations [11].

In 11 dogs (2 untreated, 4 permethrin- and 5 fipronil + S-methoprene-treated) one or more TBD-infections were detected, although owners did not report tick infestation throughout the year. It must be assumed that the owners missed several ticks, which might be explained by the variable ability of owners to search and remove ticks. Identifying ticks visually may provide a better chance of early removal when attached ticks are still small in size, whereas searching ticks by palpating the coat and skin may only result in finding engorged ticks that had already transmitted pathogens. Juvenile tick stages may have been found by some owners but not identified as ticks and therefore have been ignored. Search and removal of ticks are thus additional measures to reduce the events of transmission but cannot prevent infections completely, especially in dog owners with limited ability to find and remove ticks.

The present study provides data on the natural open field efficacy of spot on acaricides when applied by dog owners. The fact that dog owners did not apply the acaricide regularly according to the manufacture’s guidelines is certainly the major reason for the poor performance of prevention measures in this trial. Therefore the repellent and acaricidal effect of both drugs was demonstrated only by the temporary influence on tick infestation but not by any influence on infection incidence during the year. Efficacy studies on acaricidal drugs under laboratory conditions result in a maximum positive measureable effect on tick killing and prevention of pathogen transmission [12,14,30], although Estrada-Pena and Venzal Bianchi [31] also described an insufficient prevention of the transmission of *Rickettsia conorii* after application of fipronil + S-methoprene and permethrin; only amitraz was able to reduce transmission effectively. Especially the permethrin spot on, used in this trial, has been demonstrated to have a proven high efficacy in the protection against TBD transmission when administered correctly either in a regular treatment regime [13] or within the claimed efficacy period [12]. The proven efficacy of such acaricidal substance has a considerably reduced effect when applied incorrectly.

## Conclusions

Clinical disease in dogs exposed to tick-borne pathogens is rare, although a humoral immune response reflecting infection is common. Therefore a single positive antibody titer in dogs should not lead as a matter of principle to the diagnosis of a tick-borne disease. The irregular and inconsequential acaricide application by the owners demonstrates the limitation of effective protection of dogs against TBDs when this measure is left to the owner’s responsibility. The resulting, “self-inflicted” inefficacy of acaricidal applications may be the reason for the disaffection of many dog owners and veterinarians when evaluating the efficacy of acaricidal agents under natural conditions. More and intensified awareness training for dog owners, initiated by veterinarians that have to be advised on that specific problem, appears to be necessary to make the spot on application of acaricides effective regarding the prevention of tick-borne diseases. Acaricidal treatments with longer treatment intervals might overcome poor owner compliance and improve the control of tick infestations in dogs in the field.

## Competing interests

The authors declare that they have no competing interests.

## Authors’ contribution

ML participated in conception and design of the study, performed the statistical analysis, and drafted the manuscript. AF has made substantial contributions to the acquisition and analysis of data. GD has been involved in the molecular genetic studies. AJ participated in the design and coordination of the study and helped to draft the manuscript. All authors read and approved of the final manuscript.
